# Two distinct resident macrophage populations coexist in the ovary

**DOI:** 10.3389/fimmu.2022.1007711

**Published:** 2022-12-20

**Authors:** Nianyu Li, Zhuqing Li, Fang Fang, Chendi Zhu, Wenzhe Zhang, Yueshuang Lu, Rongrong Zhang, Pinxin Si, Yuehong Bian, Yingying Qin, Xue Jiao

**Affiliations:** ^1^ Center for Reproductive Medicine, Shandong University, Jinan, Shandong, China; ^2^ Key Laboratory of Reproductive Endocrinology of Ministry of Education, Shandong University, Jinan, Shandong, China; ^3^ Shandong Key Laboratory of Reproductive Medicine, Jinan, Shandong, China; ^4^ Shandong Provincial Clinical Research Center for Reproductive Health, Jinan, Shandong, China; ^5^ Shandong Technology Innovation Center for Reproductive Health, Jinan, Shandong, China; ^6^ National Research Center for Assisted Reproductive Technology and Reproductive Genetics, Shandong University, Jinan, Shandong, China; ^7^ Suzhou Institute of Shandong University, Suzhou, Jiangsu, China

**Keywords:** macrophages, ovary, phenotype, tissue-resident, function

## Abstract

**Introduction:**

Tissue-resident macrophages (TRMs) are highly heterogeneous and have a complex and important role in tissue support, homeostasis, and function. The heterogeneity, maintenance, and function of TRMs, as one of the major immune cells in the ovary, are not well understood.

**Methods:**

Application of flow cytometry, Parabiosis, Fate mapping, Macrophage depletion, etc.

**Results:**

Here, we described two distinct macrophage subsets, F4/80^hi^CD11b^int^ and F4/80^int^CD11b^hi^, with different phenotypic characteristics in the ovary of mice. The F4/80^hi^CD11b^int^ population contained a distinct CD206^+^ subgroup and highly expressed CD81, while the F4/80^int^CD11b^hi^ subset showed higher expression of CCR2 and TLR2. Notably, Ly6c^+^ macrophages were present almost exclusively in the F4/80^int^CD11b^hi^ subpopulation. Combining in vivo fate mapping and parabiotic mouse models, we characterized the longevity and replenishment of the two macrophage populations. We found that both the F4/80^hi^CD11b^int^ and F4/80^int^CD11b^hi^ subsets were ovary-resident. Importantly, the F4/80^hi^CD11b^int^ macrophages acted as a self-maintaining and long-lived population with a modest monocyte contribution at a steady state, and the F4/80^int^CD11b^hi^ subpopulation had a relatively short lifespan with a greater contribution from monocytes. After macrophage ablation, disturbance of estradiol secretion and ovarian hemorrhage due to damaged vascular integrity was observed in mice.

**Discussion:**

Our data provide critical insights into ovarian macrophage heterogeneity and highlight the strategic role of TRMs in ovarian homeostasis and physiology.

## 1 Introduction

Macrophages are important effector cells of the innate immune system that phagocytose and degrade necrotic cells, debris, and foreign bodies, coordinate the immune response and play a critical role in host defense. These cells are also involved in tissue homeostasis, remodeling, and immune surveillance (1–3).

In recent years, tissue-resident macrophages (TRMs) have been identified in nonimmune-specific organs, they settle in certain niches and have the capacity for self-renewal. With the establishment of fate mapping models and the development of single-cell sequencing technology, the unique origin, phenotypic and functional characteristics of TRMs are becoming clear and gaining attention. For example, pulmonary TRMs originate from embryonic yolk sacs and hematopoietic stem cells and are widely distributed in the alveoli and interstitium, functioning to maintain lung barrier immunity and showing functional specificity at different ages and with different microbial exposures or pathological states (4–6); Articular luminal TRMs are derived from embryonic hematopoietic stem cells that provide tight junctions and form an anti-inflammatory barrier to joints, and their functional defects contribute to the development of rheumatoid arthritis (7, 8). Thus, TRMs in different locations are highly heterogeneous and play an important role in maintaining specific physiological functions of their tissues and organs, and their abnormal functions may lead to the development of various diseases.

In addition to the heterogeneity among different tissues, TRMs within the same tissue can still be divided into multiple subpopulations based on different phenotypes and functions. Two interstitial TRMs populations, Lyve1^lo^MHCII^hi^ located adjacent to nerve fibers and Lyve1^hi^MHCII^lo^ subgroup near blood vessels, have been found to exist across multiple tissues, including the lung, heart, fat, and dermis (9). In the testis, there were CD64^hi^MHCII^−^ interstitial macrophage populations with a spherical shape and CD64^lo^MHCII^+^ peritubular populations with an elongated morphology (10, 11). Lever et al. (12) identified two subpopulations of macrophages (F4/80^hi^ macrophages and F4/80^low^ macrophages) with differential dynamics and varying degrees of monocyte contribution in kidneys.

In the ovary, macrophages have been considered the most abundant immune cells (13). They were densely distributed in the degenerating corpus luteum and atretic follicles, with sparse to moderate presence in the thecal layer of developing follicles (14–16). Macrophages exhibited quantity variations during different estrous cycles, as the number of ovarian macrophages appears to change with ovulation (13). The specific localization pattern and dynamics of macrophages in the ovary indicate their involvement in ovarian homeostasis. In comparison with other TRMs, little is known about the developmental, phenotypical, and functional heterogeneity of TRMs in the ovary. In 2020, Jokela et al. (17) employed the single-cell mass cytometry (CyTOF) to reveal for the first time that the mechanisms of controlling monocyte immigration and the ability of fetal-derived macrophages to interconvert in the ovary, and they found that the fetal-derived MHCII^-^ macrophages postnatally differentiate in the maturing ovary to MHCII^+^ cells. However, the diverse phenotype of ovarian macrophages, the maintenance of ovarian macrophages in adulthood, and how they contribute to ovarian microenvironmental homeostasis remain to be further elucidated.

Here, we defined the immunological characteristics of TRMs that reside in adult mouse ovaries. Using immunophenotyping, long-term parabiosis, and fate mapping analysis, we found that the steady-state adult ovary contains two heterogeneous macrophage subpopulations, F4/80^hi^CD11b^int^ and F4/80^int^CD11b^hi^, with distinctive cell surface proteins, ovarian residency, longevity, and local characteristics. In addition, estradiol production and vascular integrity were associated with the availability of ovarian resident macrophages. Our data further refine the heterogeneous phenotypic characteristics and function of macrophages in the ovary and reveal their unique adaptations in the ovarian microenvironment.

## 2 Methods and materials

### 2.1 Mice

Female C57BL/6J CD45.2 mice (8-week-old) were obtained from Beijing Vital River Laboratory (Beijing, China). Cx3cr1^tm2.1(cre/ERT2)Litt^, Rosa26^tm14(CAG-tdTomato)Hze^, and CD45.1 mice with a C57BL/6J background were purchased from Shanghai Biomodel Organism (Shanghai, China). Mice were bred and housed under specific pathogen-free conditions in the animal facility of the Experimental Animal Center, Shandong University (EAC-SDU, Jinan, China). All animal studies were performed according to SDU guidelines for the use and care of live animals and approved by the School of Medicine, Shandong University.

### 2.2 Parabiosis

8-week-old weight-matched female congenic CD45.1 and CD45.2 mice were surgically connected in parabiosis, as previously described and in accordance with approved protocols (7). Briefly, after lateral skin incisions were made from the elbow to knee in each mouse, the forelimbs and hind limbs were tied together by polypropylene suture, and the skin incisions were close to form a continuous suture under anesthesia. Postoperative pain management with buprenex compound, 5% dextrose and 0.9% sodium chloride, nutritional gel packs in each cage, and antibiotics (Neomycin) in the drinking water for the duration of the experiment. Mice were sacrificed 2, 3, and 8 weeks after surgery to analyze chimerism in different organs.

### 2.3 Fate mapping

For the induction of Cre recombinase to trace CX3CR1^+^ ovarian macrophages, CX3CR1^CreER^ mice were crossed to R26^Tdtomato^ mice, and CX3CR1^CreER^: R26^Tdtomato^ females were administered 75 mg tamoxifen (TAM) (Sigma)/kg body weight *via* intraperitoneal injection for 4 consecutive days. The frequency of Tdtomato^+^ cells in the blood, brain, and ovary were analyzed at 1, 4, 10, and 14 weeks after TAM by flow cytometry.

### 2.4 Macrophage depletion

To deplete macrophages, The female C57BL/6J mice (8-week-old) were treated intravenously with the injection of clodronate liposomes or control liposomes (FormuMax; 50 uL on day 1, 100uL on day 4, and 7), followed by intraperitoneal administration of 0.5mg of CSF1r neutralizing antibody (clone AFS98; Bio X Cell) or control IgG (clone 2A3; Bio X Cell) on a subsequent day. Tissues were harvested on day 10 for flow cytometry analysis.

### 2.5 Cell isolation

Briefly, the mice were anesthetized and then systemically perfused with saline through the heart. Afterward, selected tissues were separated and placed in ice-PBS. The procedure for single-cell isolation as follows. Peripheral blood was collected in an anticoagulation tube to prevent coagulation. The erythrocytes from the blood, spleen, liver, and kidney samples were lysed with RBC lysis buffer (BD Biosciences). The different tissues were thoroughly minced, digested, and consecutively filtered for single‐cell suspensions. Ovary single-cell suspension was prepared essentially as described previously (18). Briefly, ovaries from 4 mice were isolated, mixed, and cut into small pieces, followed by enzymatic digestion for 20 minutes at 37°C in plain RPMI buffer (HyClone) with Collagenase IV (4 mg/ml; Gibco) and DNase (0.01 mg/ml; Sigma), Finally, the isolated cells were washed and re-suspended in PBS and filtered through a 70-µm cell strainer (BD Biosciences) to remove large cellular debris.

### 2.6 Flow cytometry

Dead cells were excluded from analysis using Zombie Aqua Fixable Viability Kit (BioLegend). The cell suspensions were pretreated by Fc-blocking with anti-mouse CD16/32 (Clone 93, Biolegend), followed by incubation with various fluorophore-conjugated antibodies for cell-surface markers in staining buffer (PBS and 1% fetal bovine serum) for 15 minutes at room temperature in the dark. The stained cells were acquired on an LSR Fortessa cell analyzer (BD Biosciences), and data were analyzed with FlowJo software (V 10.6.1, BD Biosciences).

The following antibodies were used: Zombie Aqua™ (Biolegend), CD45 (Alexa Fluor^®^ 700, 30-F11 Biolegend), F4/80 (APC, BM8, Biolegend), CD11b (Brilliant Violet 711™ and FITC, M1/70, Biolegend), MHCII (Brilliant Violet 421™ and APC-Cyanine7, M5/114.15.2, Biolegend), Lyve1 (PE, 12044382, Invitrogen), Ly6g (Brilliant Violet 650™ and Brilliant Violet 785™, 1A8, Biolegend), Ly6c (Brilliant Violet 421™ and APC-Cyanine7, HK1.4, Biolegend), Cx3cr1 (Brilliant Violet 785™, SA011F11, Biolegend), Csf1r (PE-Cyanine7, AFS98, Biolegend), CD74 (Brilliant Violet 421™, In-1, BD), CD81 (PE-Cyanine7, Eat-2, Biolegend), CD64 (Percp-Cyanine 5.5, X54-5/7.1, Biolegend), CD45.2 (Alexa Fluor^®^ 700, 104, Biolegend), CD45.1 (PE-Cyanine7, A20, Biolegend), CD206 (PE-Cyanine7, C068C2, Biolegend), CD86 (Brilliant Violet 650™, GL-1, Biolegend), CD11c (Brilliant Violet 711™, N418, Biolegend), C1q (FITC, Ma1-40313, Invitrogen), CD169 (PE-Cyanine7, 3D6.112, Biolegend), CD192 (CCR2) (Brilliant Violet 785™, SA203G11, Biolegend) and CD282 (TLR2) (PE, CB225, Biolegend).

### 2.7 Estrous cycle

After washing the vaginal introitus of mice with saline three times, the exfoliated epithelial cells were collected and smeared on glass slides. The smears were air-dried, fixed in 95% ethanol, and stained with hematoxylin-eosin (HE). The estrous stages were determined under the microscope according to the established criteria (19).

### 2.8 Immunofluorescence

The Ovarian tissues were fixed with 4% paraformaldehyde (PFA), embedded in tissue freezing medium (Leica), and frozen at −80°C. The tissues were cut into 10-µm sections using a cryostat (Leica). Slides were blocked for 30 min at room temperature with 10% Bovine Serum Albumin (BSA), followed by 0.2% Triton in PBS, and stained overnight at 4°C with the antibody F4/80 (10 μl/mL in PBS; APC, BM8, Biolegend). Hoechst 33342 (YEASEN) was used to label DNA. The fluorescent images were captured with fluorescence microscopes (Olympus).

### 2.9 EdU incorporation

Mice were injected intraperitoneally with 2mg EdU (Click-iT™ EdU Alexa Fluor™ 488 Flow Cytometry Assay Kit, Invitrogen) and killed after 24 hours. Ovaries were collected and prepared for single-cell suspensions. EdU^+^ cells were analyzed by flow cytometry according to the manufacturer’s instructions.

### 2.10 Phagocytosis

A total of 0.1mg of pHrodo™ Green E. coli BioParticles™ (Invitrogen) was administrated i.v. (via tail vein injection) to female mice of 8-week-old. Mice were injected with PBS as controls. Mice were sacrificed two hours after the injection and the ovaries were harvested for flow cytometry.

### 2.11 Serum preparation and hormones level measurement

The mice at the diestrus stage were sacrificed and blood was collected. The serum was isolated by centrifuging at 2,500 rpm at 4°C for 15 mins after incubated at 4°C overnight. The estradiol and progesterone concentration were measured by the radioimmunoassay method (Beijing North Institute of Biotechnology, China).

### 2.12 Histology and immunohistochemistry staining

Bouin-fixed, paraffin-embedded ovarian tissue was serially sectioned at 5 μm in thickness. After the HE staining, every fifth section was analyzed for the presence of oocytes and follicles as previously described (18). The counting results were multiplied by five to estimate the total number of oocytes and follicles in each ovary. For collagen staining, tissue sections were also stained with Sirius red with Picro Sirius Red Stain Kit (Maokangbio). For immunohistochemistry, the sections of mice ovaries were stained with anti-CD31 antibody (1:50, Abcam). The image was taken with the microscope (OLYMPUS) and analyzed by ImageJ software (National Institutes of Health, NIH).

### 2.13 Quantitative RT-PCR

>Total RNA was isolated using TRIzolTM reagent (Ambion), and the RNA quality was measured by agarose gel electrophoresis. cDNA was generated with a PrimeScript RT Reagent Kit (Takara Bio). Quantitative real-time PCR was performed in triplicate using SYBR^®^ Premix Ex TaqTM Kit (Takara Bio) on Roche LightCycle^®^ 480 (Roche). The level of target gene expression was quantified after normalization to *Gapdh* expression.

Primer sequences (20–26): *Tnfα* forward primer: CGGGCAGGTCTACTTTGGAG, reverse primer: ACCCTGAGCCATAATCCCCT; *Il6* forward primer: CTTCTTGGGACTGATGCTGGT, reverse primer: CTCTGTGAAGTCTCCTCTCCG; *Fizz* forward primer: AACTGCCTGTGCTTACTCGT, reverse primer: CAAGAAGCAGGGTAAATGGGC; *Il10* forward primer: GCTCTTGCACTACCAAAGCC, reverse primer: CTGCTGATCCTCATGCCAGT; *Col1a1* forward primer: CGATGGATTCCCGTTCGAG, reverse primer: GAGGCCTCGGTGGACATTAG; *Col4a1* forward primer: GCGTAAGTTCAGCACCATGC, reverse primer: CACAAACCGCACACCTGCTA; *Vegfa* forward primer: GCACATAGAGAGAATGAGCTTCC, reverse primer: CTCCGCTCTGAACAAGGCT; *Tgfb* forward primer: CTTCAATACGTCAGACATTCGGG, reverse primer: GTAACGCCAGGAATTGTTGCTA; *Gapdh* forward primer: TGTCTCCTGCGACTTCAACA, reverse primer: GGTGGTCCAGGGTTTCTTACT.

### 2.14 Statistical analysis

All experiments were independently repeated three times or performed in triplicate. Statistical analyses were carried out using GraphPad Prism software version 8 (GraphPad Software Inc). Data were shown as Mean ± standard error of the mean (SEM), and compared with Student’s t test or Mann–Whitney tests between two groups and by one-way analysis of variance (ANOVA) among multiple groups. P < 0.05 was considered statistically significant.

## 3 Results

### 3.1 Abundance and localization of macrophages within the adult ovary

**Figure 1:**
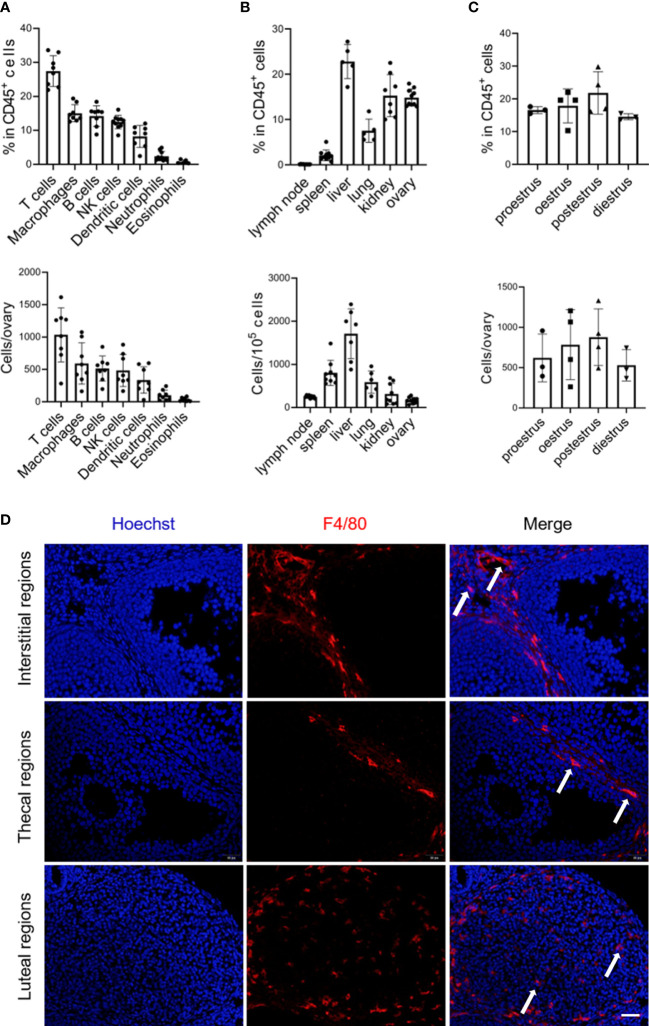
Abundance and localization of macrophages within the adult ovary. **(A)** Graphs show the proportion and number of immune cell subsets, derived from flow cytometry in **Figure S1** (n=8. Mean ± SEM). **(B)** Flow cytometry analysis of macrophage percentages and numbers in the mouse lymph node, spleen, liver, lung, kidney, and ovary (n=5~9. Mean ± SEM). **(C)** The frequencies and numbers of ovarian macrophages at the different periods of the mouse estrous cycle, each dot represents data from 4 individual mice pooled together (n=3~4. Mean ± SEM). **(D)** Immunofluorescent detection of F4/80 in the ovaries of 8-week-old mice. Arrows show ovarian macrophages. Scale bar 20μm.

To characterize the macrophage populations in the ovaries of female mice at 8 to 10 weeks of age by flow cytometry, we first gated live CD45^+^ cells to analyze the presence of the macrophage population (gating strategy and representative flow plots see **Figure S1**). Macrophages (F4/80^+^ CD11b^+^) were readily identified and constituted ~15% of the CD45^+^ immune cells in the ovary, representing the second largest population following the predominant T cells (**Figure 1A**). While although macrophages were relatively abundant in CD45^+^ immune cells within the ovary compared to the lymphoid organs (lymph nodes and spleens) and some nonlymphoid organs, such as the lungs, the absolute number was minimal in the ovary due to the different proportion of immune cells in total tissue cells (**Figures 1B**; **S2**). Within different estrous cycles, minor variations in the percentage of ovarian macrophages existed, with a trend of increase during the postestrus stage (**Figure 1C**). Next, we investigated macrophage distribution in the ovary. Given that the location of macrophages could be largely influenced by ovulation, ovaries in the diestrus period were sectioning (13). We found that F4/80^+^ macrophages were predominantly localized within theca cells, corpus luteum, and the interstitial region, but not inside follicles (**Figure 1D**).

### 3.2 Two phenotypically distinct macrophage populations reside in the ovary

**Figure 2:**
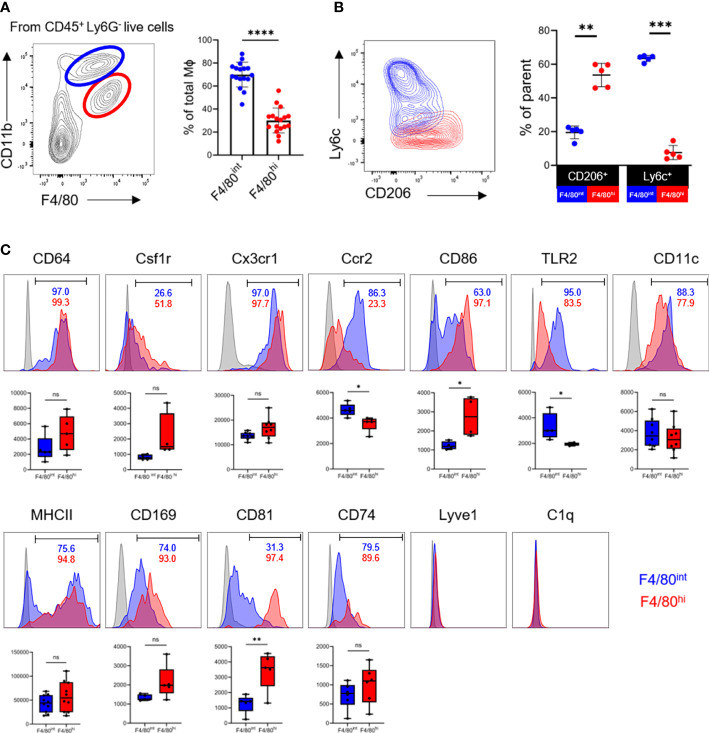
Two phenotypically distinct macrophage populations reside in the ovary. **(A)** The contour plot depicts the gating strategy for macrophages subsets and graphs show the proportion of F4/80^int^CD11b^hi^ (blue) and F4/80^hi^CD11b^int^ (red) macrophage populations in the ovaries (n=17. Mean ± SEM). **(B)** Expression of Ly6c and CD206 in two subpopulations of macrophages (n=5. Mean ± SEM). **(C)** Representative histogram, positivity ratios and mean fluorescence intensity (MFI) of macrophage surface markers expression. ns, no significance, *P < 0.05, **P < 0.01, ***P < 0.001, ****P < 0.0001.

We next asked whether the two macrophage populations from the ovary display distinct but unique phenotypes. Based on heterogeneity in the expression of canonical markers of macrophages F4/80 and CD11b, there were two subpopulations of the macrophages in the ovary, the F4/80^hi^CD11b^int^ and F4/80^int^CD11b^hi^ subsets, as shown in the murine kidney (12, 27). The F4/80^int^CD11b^hi^ macrophages significantly outnumbered the F4/80^hi^CD11b^int^ cells in the ovary (**Figure 2A**). The F4/80^hi^CD11b^int^ subset constitute the vast majority of CD206^+^ cells and were mostly Ly6C^-/low^, whereas the F4/80^int^CD11b^hi^ populations were CD206^-^ and contained a clear Ly6C^+^ subpopulation (**Figure 2B**). The interlaced expression of CD206 and Ly6C probably suggests their divergent origins (11).

Next, we investigated whether ovarian macrophages express common macrophage markers or have unique expression signatures. By flow cytometry, we found that both macrophage populations abundantly expressed CD64 (FcγR1), colony stimulating factor-1 receptor (Csf1r), and Cx3cr1. The monocyte-derived marker Ccr2 was significantly enriched in the F4/80^int^CD11b^hi^ population. Markers of pro-inflammatory (CD86, TLR2, CD11c, MHCII) and pro-repair (CD206) macrophage activation were not mutually exclusive (**Figure 2C**). In particular, the F4/80^hi^CD11b^int^ subset was essentially CD86^+^, while the F4/80^int^CD11b^hi^ population contained a fraction of CD86^-^ cells. MHCII expression was bimodal in the F4/80^int^CD11b^hi^ subgroup with both negative and positive cells, whereas it was almost positive in the F4/80^hi^CD11b^int^ cells. The coexpression of pro-inflammatory and pro-repair markers on a single cell level supports the concept of a continuous and intricate transition of macrophage polarization statuses *in vivo* in the ovary.

Several novel markers, such as CD169, CD81, CD74, Lyve1, and C1q, have recently been identified as enriched in tissue macrophages and shared by multiple organs, such as lung, kidney, and synovial tissue (27–29). We further determined the presence of these molecules in ovarian macrophages. Interestingly, CD81 was expressed predominantly in the F4/80^hi^CD11b^int^ cells but not in the F4/80^int^CD11b^hi^ populations. The percentage and mean fluorescence intensity (MFI) of CD169 and CD74 expression did not differ significantly between the two populations. Notably, Lyve1 and C1q were barely detected in ovarian macrophages, distinct from that in pulmonary and renal macrophages (9, 27) (**Figures 2C**; **S3**). Taken together, these data suggest that two phenotypically distinct macrophage populations, the F4/80^hi^CD11b^int^ and F4/80^int^CD11b^hi^ subsets, could be identified in the adult ovary in the resting state. These populations share core markers with tissue macrophages of other organs, while concomitantly expressing a unique set of indicators consistent with their organ-specific functions.

### 3.3 Tissue-resident properties of the two macrophage subsets in the ovary

**Figure 3:**
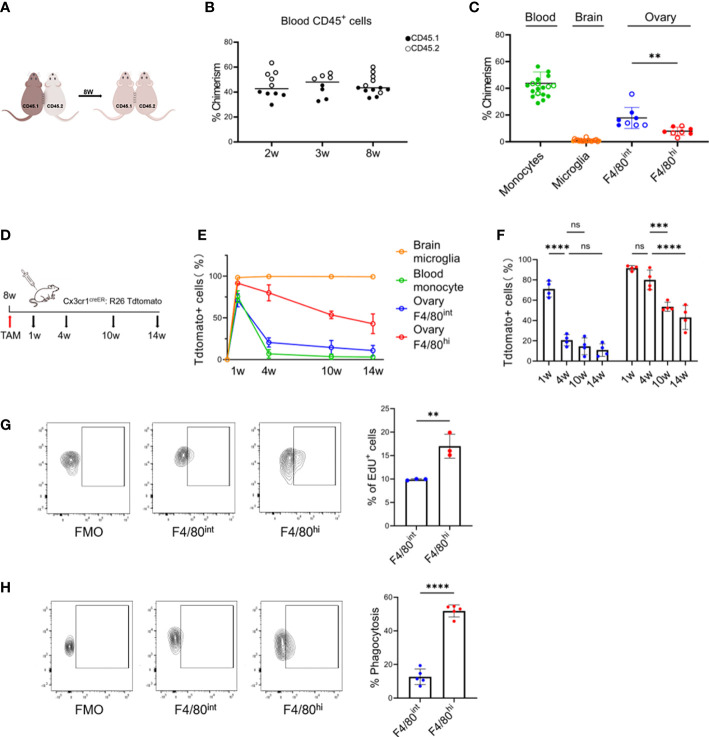
Tissue-resident and self-maintaining characteristics of the two macrophage subsets in the ovary. **(A)** Parabiosis experimental design. **(B)** Percent chimerism among host immune cells in blood from parabiotic chimeras (n=8~14, Mean ± SEM). **(C)** Chimeric ratios of monocytes, microglia, and two subpopulations of macrophages in the ovary (for the ovarian data, each dot represents data from 4 individual mice pooled together, while for the data of blood and brain, each point represents one mouse. Mean ± SEM). **(D)** Fate mapping experimental design. **(E)** Changes in the proportion of Tdtomato^+^ cells with time in the different populations. **(F)** Quantification of Tdtomato-labeling in two subsets of ovarian macrophages (n=4. Mean ± SEM). **(G)** EdU^+^ cells gating strategy and the percentage of proliferating EdU^+^ cells after 24 hours of pulse-labeling (n=3. Mean ± SEM). **(H)** Uptake of intravenously administered fluorescent E. coli BioParticles by two macrophage subpopulations in the ovary. (n=4. Mean± SEM). ns, no significance, **P < 0.01, ***P < 0.001, ****P < 0.0001.

To determine the tissue residency of the two macrophage populations in the ovary, we established parabiosis between wild-type congenic CD45.1 and CD45.2 mice and assessed the presence of nonhost cells in each parabiont 8 weeks later (**Figures 3A**; **S4A**). In peripheral blood, ~44% (43.7 ± 8.6) of the major leukocyte populations were of donor origin 2 weeks after the parabiosis surgery, indicating the efficient establishment of chimeras (**Figure 3B**). Importantly, the state of parabiosis did not result in detectable changes in ovarian morphology or structure (**Figure S4B**). After 8 weeks, we measured the proportion of donor-derived cells within the monocyte/macrophage populations of the host blood, brain, and ovary. Although monocytes had achieved half host/donor chimerism, relative contributions of donor-derived CD45.1^+^ cells to the host ovarian macrophage populations were variable, 17.9% within F4/80^int^CD11b^hi^ and 7.4% within F4/80^hi^CD11b^int^ macrophages (**Figure 3C**). These results indicate that peripheral circulation contributes partially to both resident subpopulations of ovarian macrophages, especially to the F4/80^int^CD11b^hi^ population.

### 3.4 Self-maintaining characteristics of the two macrophage subsets in the ovary

Next, we determined the turnover rate of the two macrophage populations once they were stably established in the adult ovary. As both F4/80^hi^CD11b^int^ and F4/80^int^CD11b^hi^ populations were CX3CR1 positive, we treated Cx3cr1^CreER^: R26^Tdtomato^ fate-mapping mice with TAM to pulse label both populations in adult mice at 8 weeks of age and investigated the contribution of Tdtomato^+^ cells to both ovarian macrophage populations over time (**Figure 3D**). Microglia in the brain and monocytes in the blood were used as the positive and negative controls, respectively. Administration of TAM highly labeled microglia in the brain (~100%) and F4/80^hi^CD11b^int^ ovarian macrophages (~90%), and by 14 weeks approximately 43% of the F4/80^hi^CD11b^int^ ovarian macrophages expressed Tdtomato^+^. While the ovarian F4/80^int^CD11b^hi^ populations were less efficiently labeled (~70%), the percentage of Tdtomato^+^ cells decreased significantly from 4 weeks onward, more similar to what was observed in blood monocytes (**Figures 3E, F**). In addition, we measured EdU incorporation to determine the proliferation rate of ovarian macrophages under steady-state conditions. We found that EdU pulses labeled ~10% (9.9% ± 0.2%) and ~17% (17.0% ± 2.6%) of the cells in the F4/80^int^CD11b^hi^ and F4/80^hi^CD11b^int^ populations, respectively (**Figure 3G**). Taken together, our data suggest that the two subpopulations of cells have differences in turnover properties, where F4/80^hi^CD11b^int^ populations are long-lived, with a greater capacity for self-renewal.

Moreover, we also determined the scavenging function of different ovarian macrophage populations *in vivo*. The E. coli BioParticles were injected intravenously into 8-week-old mice. After a two-hour circulation time, quantitative flow cytometry analysis found that the F4/80^hi^CD11b^int^ populations showed a greater *in vivo* capacity to ingest bacterial cargoes than the F4/80^int^CD11b^hi^ cells (51.8% vs. 12.7%, p<0.0001) (**Figure 3H**), supporting their genuine macrophage nature and also indicating that functional differences exist between the two subsets of macrophages.

### 3.5 Disturbance of estradiol production and angiogenesis by macrophage depletion

**Figure 4:**
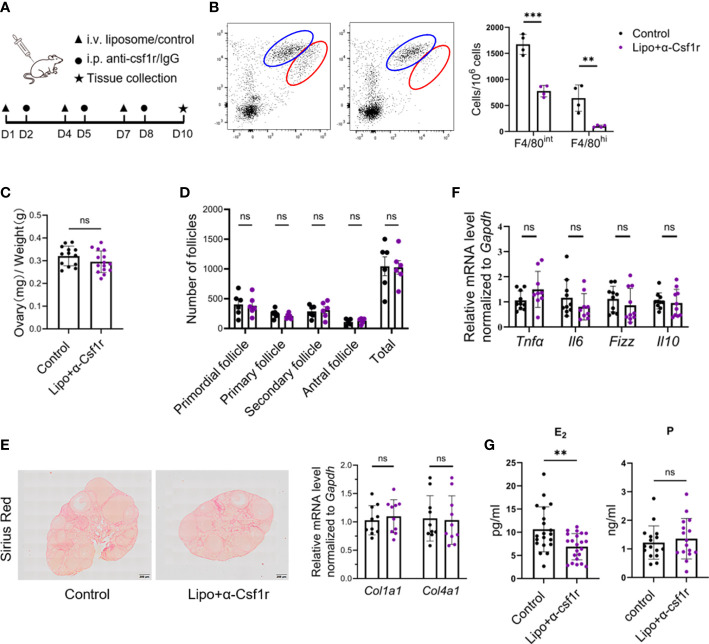
Disturbance of estradiol production by macrophage depletion. **(A)** Macrophage depletion experimental design. **(B)** Dot plots and histograms depict the two subsets of ovarian macrophages in clodronate+anti-CSF1r (lipo+αCSF1r) treated and control antibody-treated (control) mice 48 h after the last treatment (n=4. Mean ± SEM). **(C)** Ovarian index of lipo+αCSF1r treated mice and control mice (n=13~14. Mean ± SEM). **(D)** Follicle counts in lipo+αCSF1r treated mice and control mice (n=6. Mean ± SEM). **(E)** Sirius red and quantitative RT-PCR analysis of *Col1a1* and *Col4a1* in the ovaries of lipo+αCSF1r treated mice and control mice. Scale bar 200μm. **(F)** Quantitative RT-PCR analysis of proinflammatory- and anti-inflammatory-related genes in the ovaries of two groups of mice (n=9. Mean ± SEM). **(G)** Estradiol (E_2_) and progesterone (P) levels in lipo+αCSF1r treated mice and control mice (n=20. Mean ± SEM). ns, no significance, **P < 0.01, ***P < 0.001.

**Figure 5:**
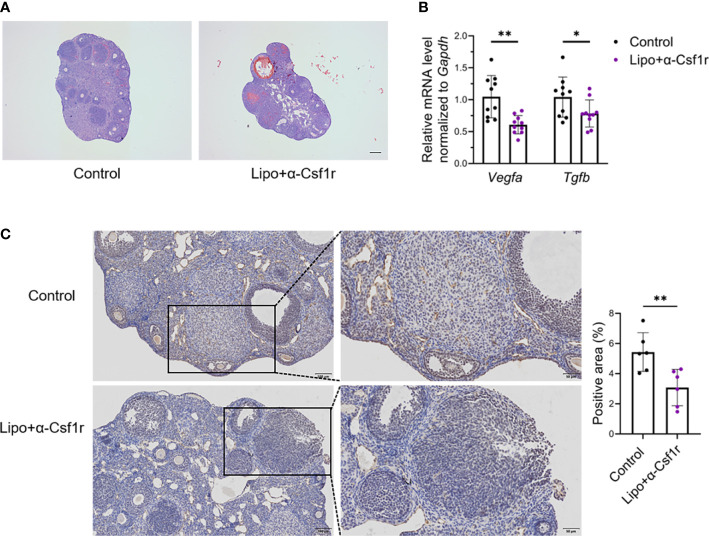
Macrophage depletion suppresses angiogenesis and induces ovarian hemorrhage. **(A)** Hematoxylin-eosin (HE) staining of lipo+αCSF1r treated mice and control mice ovaries. Scale bar 200μm. **(B)** *Vegfa* and *Tgfb* expression in the ovaries of lipo+αCSF1r treated mice and control mice (n=10. Mean ± SEM). **(C)** Immunohistochemistry and quantification of anti-CD31 in the ovaries of lipo+αCSF1 treated mice and control mice (n=6. Mean ± SEM). Scale bar 100μm or 50μm. *P < 0.05, **P < 0.01.

To gain insight into the importance of ovarian macrophages for normal ovarian function, we depleted macrophages with clodronate liposome and anti-CSF1r antibody treatment using a previously described dosing regimen (17, 30, 31) (**Figure 4A**). Short-term macrophage depletion resulted in a loss of two-thirds of macrophages in the ovary without affecting other immune cells (**Figures 4B**; **S5**). We did not find significant differences in ovarian size, follicle counts, or fibrotic indices between the macrophage-depleted and control mice (**Figures 4C–E**). Macrophage depletion did not significantly alter the ovarian expression of the proinflammatory- and anti-inflammatory-related genes *Tnfa*, *Il6*, *Fizz*, and *Il10* (**Figure 4F**). Of note, the levels of estradiol were markedly decreased in macrophage-depleted mice, but progesterone did not show significant changes (**Figure 4G**). In addition, the majority of ovaries in the macrophage-depletion group showed hemorrhage (**Figure 5A**). The expression of the vascular neovascularization marker *Vegfa* and angiogenesis-regulated gene *Tgfb* was found to be significantly decreased in macrophage-depleted ovaries by quantitative RT-PCR. In addition, immunochemical staining for vascular endothelial cell marker CD31 showed significantly decreased expression (**Figures 5B, C**). The above results suggest that macrophages are essential for ovarian tissue homeostasis, especially for estradiol synthesis and vascular integrity.

## 4 Discussion

As tissue-resident cells, macrophages play an important role in organ homeostasis and disease. In recent years, multiple studies have shown that TRMs in different tissues and organs are highly heterogeneous. However, little is known about the TRMs in the ovary due to the tiny size of the tissue and the few absolute numbers of immune cells in them. Here, we have characterized the phenotypes, tissue-resident characteristics, longevity, and functions of ovarian macrophages in the steady state. We defined F4/80 and CD11b as superior markers to study the two major macrophage populations in the ovary. We further showed that bone marrow-derived monocytes contribute to the macrophage pool in the adult ovary. The contribution of peripheral circulation to the long-lived subgroup F4/80^hi^CD11b^int^ was significantly weaker than that of the F4/80^int^CD11b^hi^ subgroup. Finally, macrophage depletion experiments revealed that macrophages are essential for ovarian estradiol production and vascular integrity. Collectively, these data reveal the hitherto unknown diversity of ovarian macrophages and demonstrate that two distinct populations of macrophages exist in the ovary with different immunological characteristics.

The immune atlas in the ovary has been rarely reported compared with other organs. We found that various immune cell types resided in the ovary, and macrophages represented the second largest population following the predominant T cells. While no variations were observed within different estrous cycles, although with a trend of increase during the postestrus stage. Previous studies on whether the distribution and number of macrophages change during different estrous cycles were rather limited. Petrovská et al. found that the density of macrophages distributed in different substructures of the ovary varies dynamically during the cycle by immunohistochemistry. For example, macrophage density of the thecal layer was significantly increased in the ovaries from proestrus and estrus when compared with other cycle stages (14). Brännström et al. claimed that the number of ovarian macrophages increased in the pre-ovulatory period (32). The inconsistency may be explained by different strategies used for experiments, with the physiological cycle in our study and the ovulation cycle induced by human Chorionic Gonadotrophin (hCG) by Brännström et al. Exploring the dynamics of macrophages during the cycle may help us understand the significance of the immune microenvironment during ovulation.

In addition to the developmental heterogeneity, a substantial amount of TRMs heterogeneity arises from the diversity of the tissue environments in which these cells reside. Several studies have shown that macrophage populations exhibit distinct transcriptional profiles and epigenetic markers that are specific to the tissues in which they reside (6, 33–35). These findings highlight the critical role of tissue factors in imprinting macrophage transcriptional programs and influencing macrophage development, activation, and functional diversity, with implications for disease and homeostasis. However, the heterogeneity of some macrophage populations, such as ovarian macrophages, is not fully understood. Jokela et al. (17) first proposed a level of heterogeneity in ovarian macrophages by identifying three phenotypically distinct subpopulations of ovarian macrophages based on MHCII expression. Here, we not only verified the heterogeneous phenotypes of macrophages induced by the ovarian microenvironment in adult mice, but also divided ovarian resident macrophages into two subpopulations, the F4/80^hi^CD11b^int^ and F4/80^int^CD11b^hi^ subsets, and compared the phenotypic differences between the two subpopulations. In contrast to various other tissues, such as the heart, fat, and epidermis, where two subpopulations of Lyve1^hi^ and lyve1^lo^ TRMs were found (9), ovarian macrophages did not express Lyve1. We provide evidence that F4/80^hi^CD11b^int^ contains a higher proportion of CD206^+^ macrophages, analogous to renal macrophages, with high expression of CD81 (27). Ly6c^+^ macrophages are present in the F4/80^int^CD11b^hi^ population, suggesting that this subpopulation of macrophages is more closely related to hematopoietic stem cell-derived monocytes. We have not explored their origins, so the molecular cues driving the development of these two macrophage subpopulation lineages remain to be determined. Determination of whether an intrinsic imprinting program exists before tissue entry or whether tissue cues encountered after recruitment are major determinants of macrophage fate would be of interest. Furthermore, since the limited number of indicators can be detected simultaneously by flow cytometry, it was difficult to delve into the macrophage phenotypes profile with our current data. To gain the full breadth of marker expression in more depth, further studies applying high-throughput multiomic profiling of immune diversity are warranted.

Investigations of the ontogeny and lifespan of different macrophage populations have shown that TRMs can be derived from embryonic populations and self-sustain within tissues (3, 17, 36, 37). These studies also found that the turnover rate of adult TRMs can vary depending on the tissue site. Recent studies have shown that macrophages in the ovary are initially seeded by embryonic precursors, which are maintained into adulthood, followed by monocyte-dependent stages of macrophage seeding into the ovary, and these macrophages are maintained into adulthood. Furthermore, the authors demonstrated that embryonic-derived populations are associated with high F4/80 expression (17). Interestingly, we found that the F4/80^hi^CD11b^int^ population also exhibits more independence from the peripheral circulation and has a longer lifespan than the F4/80^int^CD11b^hi^ subpopulation, which further supports the existence of distinct resident macrophage populations in the ovary. Furthermore, we found that the F4/80^hi^CD11b^int^ population has a greater capacity for self-renewal based on the analysis of EdU integration. Studies of ovarian resident macrophages showed that the majority of F4/80^hi^CD11b^int^ macrophages were maintained during the 8-week parabiosis experiment, suggesting a relatively slow turnover of this population from the blood. Therefore, this population is mainly a combination of embryonic origin and self-renewing macrophages. Zhang et al. (38) divided the F4/80^+^CD11b^+^ ovarian macrophages into Ly6c^+^ monocyte-derived macrophages and Ly6c^-^ resident subsets, and the latter showed a significant decrease in the ovaries of aged mice. Given that the F4/80^hi^ macrophage subpopulation in our study mainly showed a Ly6c^-/low^ phenotype, there might be a decrease in the F4/80^hi^ subpopulation in ovarian aging. Further research on the fate process and dynamic differences between these two subsets during ovarian aging are warranted.

Limited by the lack of tools to specifically remove a subpopulation of macrophages, we used a holistic removal strategy to explore the unique effects of ovarian macrophages on ovarian reproductive function. Several studies have shown that the immune system is important for ovarian health (39–41). However, previous studies on the impact of macrophages on ovarian function are controversial, with some suggesting that macrophage clearance can affect ovarian physiological function (42, 43), while others suggest that a 50-60% reduction in monocytes/macrophages had no significant effect on follicles in adult ovaries, either in the short or long term (44). Similarly, we did not observe any change in the degree of follicular atresia or apoptosis in mice for 10 days after the depletion of macrophages. Nevertheless, we found disturbed estradiol levels in the mice, suggesting that depletion of macrophages disturbed the immune microenvironment of the ovary and affected ovarian function, but no significant change in follicle number was found, indicating compensatory maintenance of the body in response to immune cell turmoil. However, the normal number does not equal to the normal quality, and therefore the effect on oocyte quality remains to be explored. Furthermore, the potential effects of short-term treatment may be relatively weak, thus whether long-term macrophage depletion has a more profound effect on reproductive function deserves further investigation.

Recent findings have identified various subsets of TRMs and their contribution to vascular system development. For instance, Leid et al. (45) have characterized the dedication of embryonic macrophages in remodeling the developing coronary vascular plexus. In this context, we found hemorrhage and disturbed angiogenesis in mouse ovaries after the deletion of macrophages, consistent with what Ono et al. (43) and Turner et al. (46) reported, all of which suggest that macrophages play an important role in the structural and functional integrity of blood vessels (47).

In conclusion, this study demonstrates that two heterogeneous macrophage populations resided in the ovary play an important role in ovarian functionality with different phenotypes, residential properties, longevities, and functions. Our data provide functional insights to better understand the biology of ovarian macrophages at a steady state and expand the understanding of the immunological properties of the female reproductive system region.

## Data availability statement

The original contributions presented in the study are included in the article/**Supplementary Material**. Further inquiries can be directed to the corresponding author.

## Ethics statement

The animal study was reviewed and approved by Institutional Animal Care and Use Committee at Shandong University.

## Author contributions

XJ and NL conceived and designed the experiments. NL, ZL, FF, CZ, WZ, YL, RZ, PS, and YB performed the experiments. XJ and NL analyzed the data and wrote the paper. YQ and XJ funded and supervised the research. All authors contributed to the article and approved the submitted version.
