# Supplementary material for: Two distinct resident macrophage populations coexist in the ovary
Source: Front Immunol. 2022 Dec 20;13:1007711. doi: 10.3389/fimmu.2022.1007711 (PMC9810109; doi:10.3389/fimmu.2022.1007711)
Supplement: Supplementary file 1 [file DataSheet_1.pdf]

Supplementary Material

1 Supplementary Figures

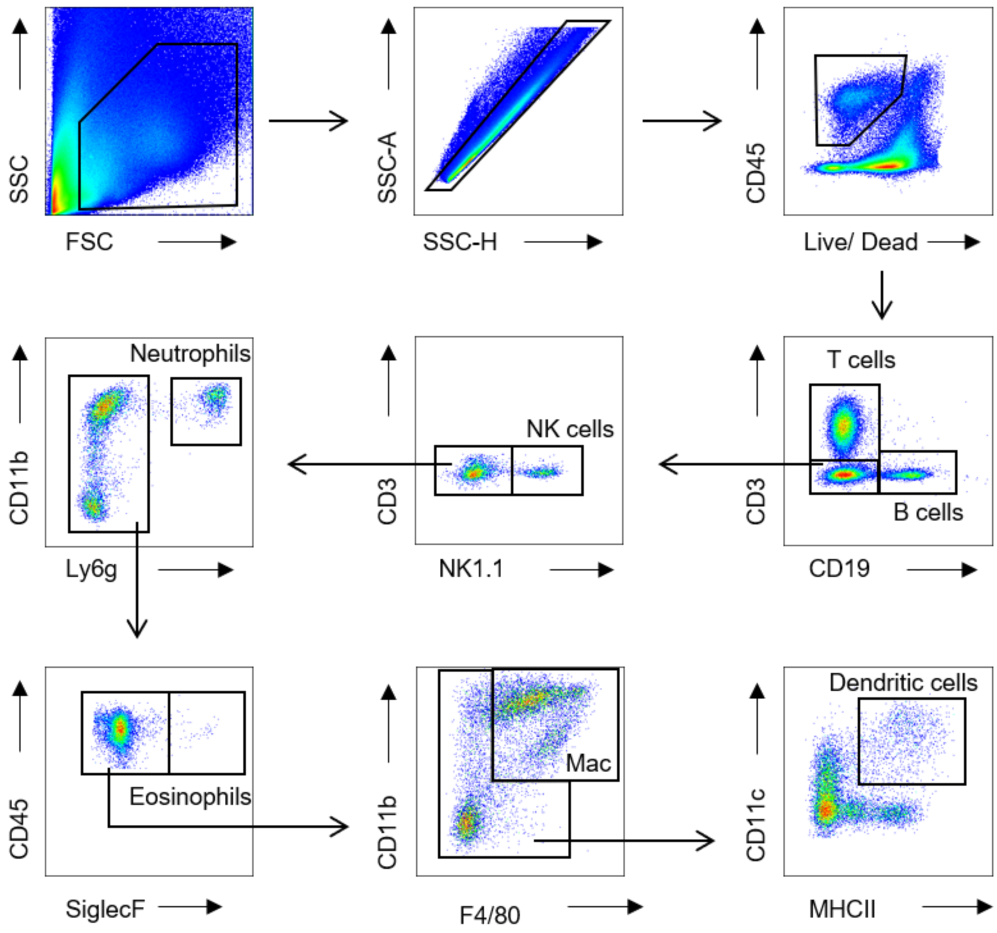

Supplementary Figure 1. Gating strategy of immune cells in the ovary. Mac, macrophages.

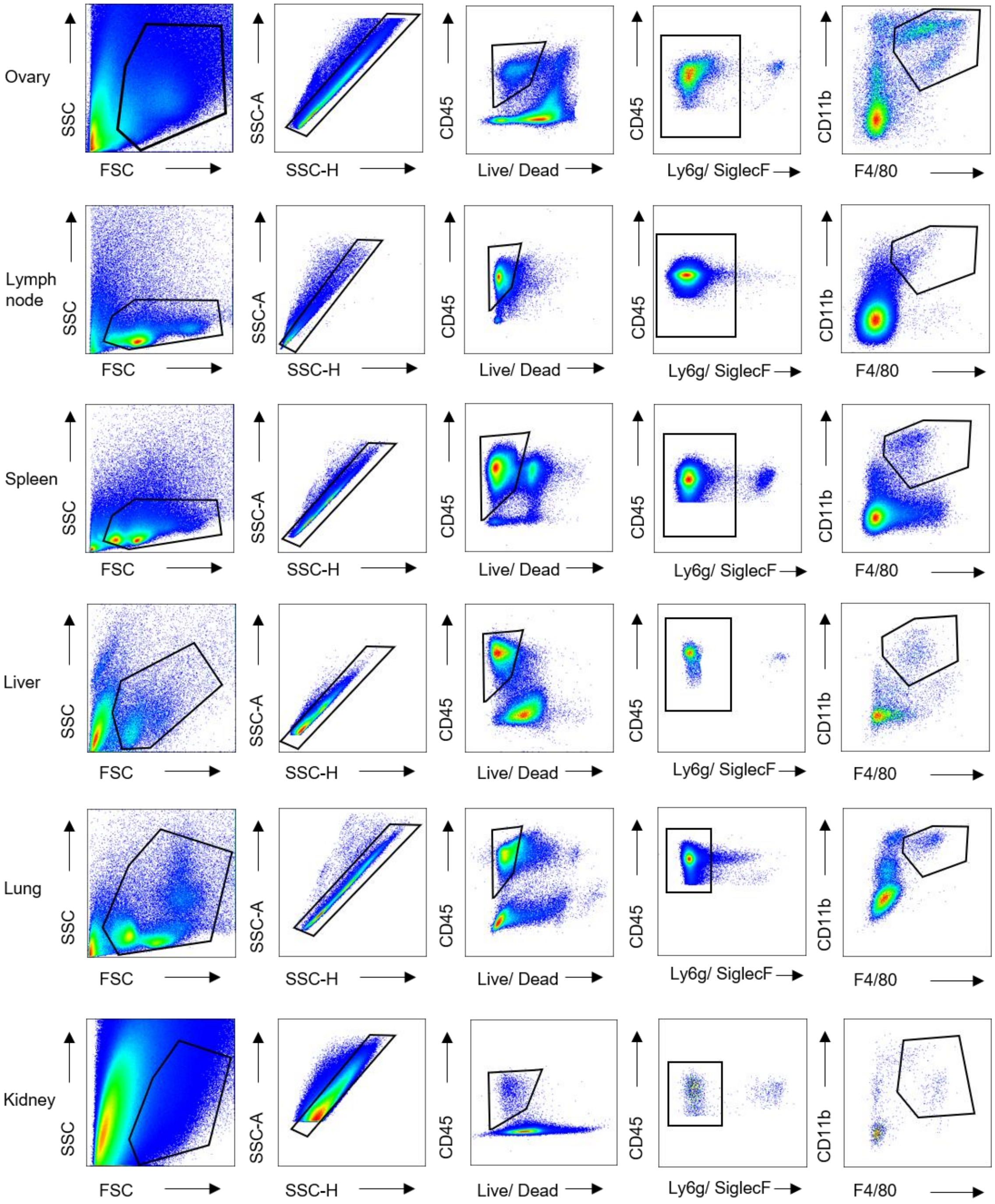

Supplementary Figure 2. Gating strategy of macrophages in the ovary, lymph node, spleen, liver, lung, and kidney.

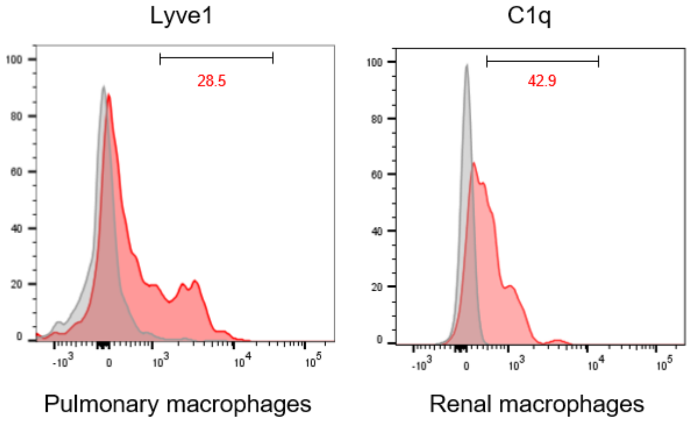

Supplementary Figure 3. Representative histograms and positivity ratios of Lyve1 and C1q labeling in pulmonary macrophages and renal macrophages, respectively.

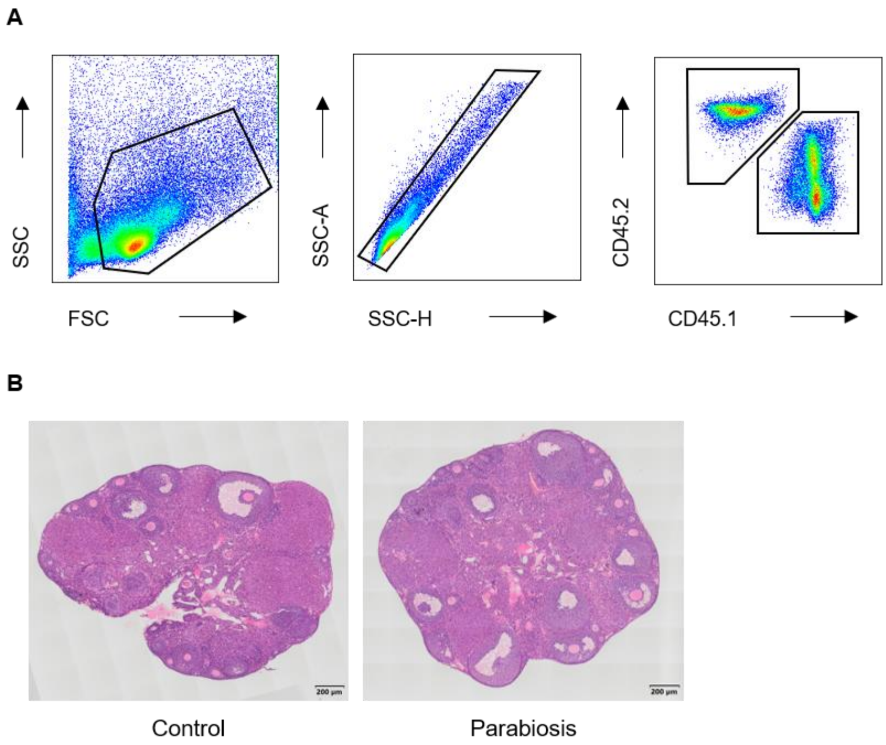

Supplementary Figure 4. Gating strategy and ovarian changes in parabiotic chimeras. (A) Gating strategy of chimerism. (B) Hematoxylin-eosin (HE) staining of parabiosis mice and control mice ovaries. Scale bar 200μm.

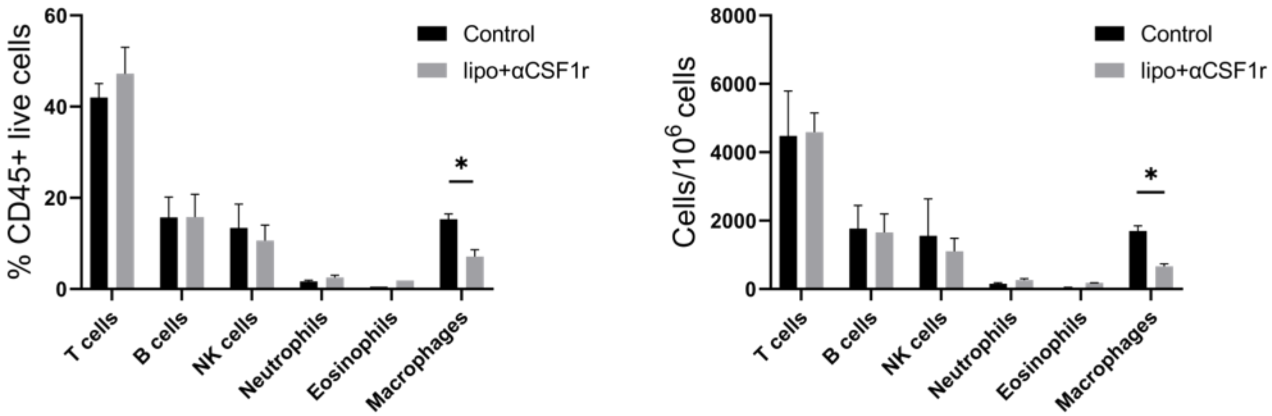

Supplementary Figure 5. The frequencies and numbers of different immune cell types in the ovary of control and lipo+αCSF1r treated mice (n=3. Mean ± SEM). *P < 0.05.
